# Stratification of non-small cell lung cancer patients for therapy with epidermal growth factor receptor inhibitors: the EGFR fluorescence in situ hybridization assay

**DOI:** 10.1186/1746-1596-1-19

**Published:** 2006-08-15

**Authors:** Marileila Varella-Garcia

**Affiliations:** 1 University of Colorado Health Sciences Center, Cancer Center, Aurora, CO, USA

## Abstract

DNA fluorescence in situ hybridization (FISH) technology is used to study chromosomal and genomic changes in fixed cell suspensions and tissue block preparations. The technique is based on specific hybridization of small labeled DNA fragments, the probes, to complementary sequences in a target DNA molecule. Demand for FISH assays in formalin-fixed, paraffin-embedded tissues has been increasing, mainly in conditions in which diagnosis is not achieved in cell smears or tissue imprints, such as solid tumors. Moreover, the development of molecular targeted therapies in oncology has expanded the applicability of tests to predict sensitivity or resistance to these agents. The efficient use of tyrosine kinase inhibitors (TKI) of the epidermal growth factor receptor (EGFR) as therapeutical agents in advanced non-small cell lung cancer (NSCLC) depends on identification of patients likely to show clinical benefit from these specific treatments. The EGFR gene copy number determined by FISH has been demonstrated as an effective predictor of outcome from NSCLC patients to EGFR TKIs; however there are pending challenges for standardization of laboratory procedures and definition of the scoring system. This methodology article focuses on the EGFR FISH assay. It details the scoring system used in the studies conducted at the University of Colorado Cancer Center in which a significant association was found between increased EGFR copy numbers and clinical outcome to TKIs, and proposes interpretative guidelines for molecular stratification of NSCLC patients for TKI therapy.

## Predictive markers in carcinomas

Improving survival of cancer patients has been a difficult task for oncologists and all other related medical specialists due to the complexity of this group of diseases. Prevention, early detection and improvement in therapeutic options are the major approaches that can make a difference and have received outstanding attention from cancer physicians, researchers and funding agencies. Much has been learned in the last decade about tumor biology and genetics, and a better understanding of cellular mechanisms underlying the initiation and progression of cancer has enabled the development of innovative therapeutical strategies. Among these are the molecular-based therapies, which address specific cell signaling pathways that are important tumor-drivers.

The molecular targeted therapy field is still in its early stages of exploration. However, exciting results have been reported including examples of dramatic improvement in outcome for neoplasias previously known for their poor prognosis. One of the first validated targeted therapies in oncology involves metastatic breast cancer and the monoclonal antibody trastuzumab (Herceptin, Genentech Inc, San Francisco CA) [1]. In approximately 20% of breast cancers the human epidermal growth factor receptor 2 gene (c-erb-B2, ERRB2 or HER2), a member of the receptor tyrosine kinase 1 (RTK1) family, is amplified and overexpressed at the receptor level and these tumor characteristics are significantly associated with poor clinical outcome [2]. However, women with HER2 overexpressing metastatic breast cancer received a significant benefit from trastuzumab, a recombinant humanized monoclonal antibody launched as a therapeutic option in 1998. The selection of these patients for treatment has been made by evaluating the levels of protein expression in immunohistochemical assays (IHC) and/or the number of copies of the HER2 gene in fluorescence in situ hybridization assays (FISH) [3,4].

More recently, one international (HERA) and two NCI-sponsored phase III clinical trials (NSABP B31 and NCCTG N9831), which have enrolled more than 6,000 patients, have shown that combining paclitaxel with trastuzumab after adjuvant chemotherapy significantly improved outcome among women with surgically removed HER-2 positive breast cancer [5,6]. These results expanded the spectrum of breast cancer patients potentially eligible for trastuzumab therapy from metastatic to early stage breast cancer.

Non-small cell lung cancer (NSCLC) is another solid tumor which has seen a favorable impact from targeted therapy. Lung cancer is a significant public health problem in western countries and has long been the most common cause of cancer death [7]. NSCLC is usually diagnosed in advanced stage, when prognosis is poor and options for chemotherapy are limited. Another member of the RTK1 family, the epidermal growth factor receptor (EGFR, HER1), is long known to be overexpressed in a significant fraction of NSCLC [8]. EGFR is a 170 kDa type I growth factor membrane receptor with 1186 amino acids encoded by 28 exons spanning near 190 kb on chromosome 7p11.2. These receptors exist as active monomers but, upon binding to ligands such as the epidermal growth factor (EGF) and the transforming growth factor alpha (TGFα), they undergo conformational changes that facilitate dimerization, either with another EGFR molecule or with HER2, HER3 or HER4 molecules. The dimerization is followed by intermolecular autophosphorylation of key tyrosine residues in the activation loop of catalytic tyrosine kinase domains through the transfer of phosphates from adenosine triphosphate (ATP). EGFR-activated pathways include the mitogen-activated protein kinase (MAPK) pathway, which induces cell proliferation, as well as the AKT and the signal transducer and activator of transcription (STAT) pathways, which contribute to cell survival.

The role of EGFR as an oncogene has been elucidated for many years and the level of EGFR protein expression has been shown to be elevated in multiple cancer types relative to normal tissues [9]. In lung cancer, there are several key mechanisms for EGFR activation, such as overexpression of ligands [10], gene amplification [11,12] and activating mutations [13]. The discovery of agents with the ability to antagonize EGFR functions in cancer cells, such as the monoclonal antibody cetuximab (Erbitux, ImClone Systems Inc) and the specific tyrosine kinase inhibitors (TKIs) gefitinib (Iressa, AstraZeneca, UK) and erlotinib (Tarceva, OSI Pharmaceuticals Inc) have heightened the clinical interest in this growth factor receptor (14,15). Gefitinib and erlotinib are orally active EGFR-TKIs that block signal transduction pathways implicated in the proliferation and survival of cancer cells and other host-dependent processes promoting cancer cell growth. These drugs have intracellular mechanisms of action and compete with ATP for binding to EGFR thus directly inhibiting EGFR autophosphorylation. In retrospective studies, patients with advanced NSCLC treated with gefitinib or erlotinib have shown higher rates of objective response and longer survival than non-treated patients [16,17]. Erlotinib was shown in the NCIC-BR.21 phase III trial to provide significant survival benefit to NSCLC patients compared with placebo [18] and was approved for clinical use in the US (November 2004) and Europe (June 2005). Gefitinib failed to reach significant improvement in survival in the large randomized ISEL (Iressa Survival Evaluation in Lung Cancer) study [19]. Nevertheless, there was a clear trend towards better outcome in the treated group of patients comparing to placebo, suggesting that there is a subset of patients who benefit from using gefitinib. Interestingly, in both erlotinib and gefitinib NSCLC randomized trials approximately 30% of the patients died within 4 months of treatment time, which strongly suggest that there is a subset of lung cancer patients who receive no clinical benefit from EGFR-TKI therapy.

The availability of molecular targeted therapy agents with significant anti-tumor activity in advanced NSCLC both as a single agent and in combination with chemotherapy and the need to increase the efficiency of these treatments are factors urging the development and validation of laboratory tests for assessment of the EGFR status in lung cancer patients. Research efforts have focused on a large number of clinical and biological factors that may predict which NSCLC patients could benefit from molecular targeted treatments and ultimately aimed to the development of sensitive and specific laboratorial tests to screen patients. Early on, the expression levels of EGFR was tested without a predictive success for response to gefitinib [2022], although more recent studies in larger phase II cohorts have detected a positive association between high EGFR protein levels and sensitivity to both gefitinib and erlotinib [23,24]. Activating missense mutations and deletions in the tyrosine kinase domain of the EGFR gene were the first molecular changes clearly associated with objective response to gefitinib and erlotinib [25-27]. The impact of these EGFR mutations on overall survival, however, has varied from a strong positive correlation in some populations [28-30] to a much less marked correlation in others [23,24]. Interestingly, EGFR mutations also conferred superior outcome for chemotherapy in the TRIBUTE trial [31] and may even be prognostic markers according to recent findings that non-treated patients with mutations have longer survival [24,32].

Genomic gain for EGFR sequences detected by fluorescence in situ hybridization (FISH) has also been demonstrated as an excellent predictor of response [23,24,33] and longer survival [23,33] in large cohorts of patients treated with gefitinib and erlotinib. More recently, molecular analyses performed in patients included in the ISEL study also have shown that longer survival was correlated with high levels of EGFR genomic gain detected by FISH [34]. Interestingly, if the EGFR genomic abundance in tumors is evaluated by other techniques, such as Southern Blotting or quantitative real-time PCR, the association between increased gene copy numbers and sensitivity to TKI is non-significant or marginally significant (30,35,36). The difference probably reflects the technical characteristics of each of the methodologies. FISH is an in situ method that allows for identification of single cells in the context of the tissue architecture.

Southern Blot and PCR-based techniques are global extraction-based methods that are influenced by the dilutional effects of stroma and infiltration of reactive cells.

Mutations in the tyrosine kinase domain of the HER2 gene which were similar to those described for the EGFR gene were detected at very low frequencies in NSCLC and are probably not clinically relevant [37,38]. Conversely, high level of genomic gain for the HER2 gene detected by FISH was found to be a significant predictor of outcome to gefitinib and an enhancer of sensitivity to gefitinib in EGFR FISH positive NSCLC patients [38].

## FISH assay: principle and technical challenges

The basic technique of in situ hybridization performed on intact cytologic material was described by Gall and Pardue [39] employing radiolabeled probes. The development of fluorescent labeling of nucleic acid probes [40] not only abrogated the need for radioactive reagents but also expanded the applicability of the technique allowing for simultaneous analysis of numerous chromosomal regions using distinctly labeled probes. Since then, FISH has been increasingly accepted as a powerful research tool for investigating chromosomal changes in dividing or non-dividing cells. More recently with the continuous commercial releasing of DNA probes FISH has also become an important technique in diagnostic pathology.

The key component of the FISH technology is the complementary property of the DNA molecule. After denaturation and under proper conditions of temperature and pH, the double stranded DNA molecule is accurately regenerated. If a set of single-stranded DNA fragments carrying labeled nucleotides is provided as a probe at or soon after denaturation, these fragments may be incorporated into the new double stranded molecule in specific locations determined by their nucleotide sequence. Afterwards, the fluorescent label in the probe allows for the visualization of the number of copies and location of the target sequences in interphase nuclei, chromosome spreads or DNA fibers.

In oncology, most of the biological specimens are available as formalin-fixed, paraffin-embedded (FFPE) tumor blocks. Two main technical strategies have been employed for FISH analysis of these FFPE specimens: one requires isolation of intact nuclei from thick sections of the tumor blocks and hybridization assays are performed in cell suspensions; the other utilizes thin cuts of the whole paraffin-embedded blocks for assays and analyses. In our laboratory, we favor the analysis of thin sections of whole paraffin-embedded blocks since this strategy has the critical advantage of maintaining intact the overall tissue architecture. In consequence, it is possible to perform direct comparison of the FISH results with the morphological details in sections stained by hematoxylin and eosin (H&E) or immunohistochemical stains. Moreover, in these sections it is possible to distinguish with high confidence tumor nuclei from stromal and reactive nuclei and to use the non-tumoral cells as internal controls for hybridization if necessary.

A certain level of protocol customization is required for optimum results in FISH assays performed on FFPE tissue sections. Steps specifically important are the proper handling and processing of the tissue after biopsy or resection from the patient, the removal of tissue proteins that may interfere with hybridization, and the tailoring of hybridization settings and post-hybridization washing conditions. DNA degradation and cross-linking between the DNA strands should be carefully prevented in the resected or biopsied tissue, and gross dissection of large blocks in 1–3 mm thick pieces to allow a uniform, proper fixation and incubation in 10% neutral buffered formalin for 8 to 24 hours is recommended. Degraded or cross-linked DNA prevents probe binding whereas denatured proteins generate high fluorescence noise, and in both of these situations the fluorescent signals are not detected or impossible to score. In addition, adequate fixation assures the preservation of the tissue morphology, also a critical parameter for FISH analysis in solid tumors.

Some pathology laboratories have been reluctant to embrace routine FISH testing. The main alleged disadvantages of using FISH in clinical diagnosis are the need for expensive fluorescence microscopes equipped with high quality immersion objectives and multiple fluorescence filters. Moreover, because the fluorescent signals may fade under bright illumination and fade upon extended storage, the hybridization results must be permanently recorded with digital cameras. Considering laboratory personnel, there is also a negative impact from the fact that the protocol is laborious, microscope analysis requires specialized training and there is an insufficient labor force to absorb incremental testing. However, these factors are surely improving over time. Cost of fluorescence microscopes and imaging systems are decreasing and automation of many laboratorial and analytical steps is available from more than one commercial source. Additionally, clinical laboratories both in private and in academic settings have lately seen an increase in the number of requests for FISH testing in genetic diseases, hematological neoplasias, and solid tumors. The expansion of trastuzumab therapy from metastatic to early breast cancer following recent findings that trastuzumab was effective in adjuvant settings [5,6] has already escalated the demand for HER2 FISH assays. Validation of the EGFR FISH test to select NSCLC patients for therapy with EGFR-TKIs will also bring a large number of specimens to the clinical pathology laboratories. Moreover, EGFR-TKIs are considered as treatment options in other tumors such as colorectal, head and neck and pancreatic carcinomas [14,15] and the FISH assay could prove useful for selection of these patients too. Therefore, the increasing applicability associated with factors such as the decreasing equipment costs and release of automated platforms for assaying and analyses is expected to significantly expand the community-based experience in FISH technology in the near future.

## Guidelines for the EGFR FISH assay and microscope analysis

The standard protocol for assaying and analyzing the copy number of the EGFR gene in FFPE lung sections in our laboratory using the SpectrumOrange LSI EGFR/SpectrumGreen CEP 7 probe set (Vysis/Abbott Molecular, IL) is briefly described. Typically we receive one H&E-stained and two sequential blank sections cut at 4 um and mounted on positively charged slides. Regions rich in tumor cells are identified in the H&E stained slide and the smallest area of interest necessary to cover a significant fraction of tumor is defined for hybridization and marked in the back of the blank slide with diamond-tipped scribe. The protocol is customized depending on the size of the section. The blank slide from a biopsy is incubated overnight at 56°C, while larger sections are incubated for 2 to 4 hours. Sections are initially passed through three 10-min CitriSolv (Fisher Scientific) incubations for deparaffinization, followed by 10-min immersion in 100% ethanol. Subsequently, biopsy specimens are treated with sequential incubations in 2 × SSC at 75°C for 6 min, in 0.25 mg/ml Proteinase K/2 × SSC at 37C for 10 min, and in 2 × SSC for 5 min at room temperature. Incubation times in 2 × SSC and Proteinase K are longer for larger resected specimens, going up to 20 min. Slides are then immersed in graded alcohol series (75°C, 85°C, 100°C), air-dried, and the probe is applied onto the area of interest. Based on the size and shape of this area, a specific glass coverslip is selected for each section (12 mm or 15 mm diameter, 10 × 22 mm, 18 × 18 mm, 22 × 22 mm) and the amount of probe to be used is calculated based on the area of the coverslip to maintain the final probe concentration specified by the manufacturer. The hybridization area is covered with the selected coverslip and sealed with rubber cement. Specimens are heated in a dry oven for 10–15 min at 80°C for denaturation of the target and probe DNAs and are subsequently incubated overnight at 37°C in humidified chamber. Upon completion of the hybridization time, the rubber cement seal is stripped off, the coverslip is gently removed, and the slides are immediately incubated in 2 × SCC/0.3% NP-40 at 73°C for 2 min. Alternatively, 1.5 M urea/0.1 × SSC at 45°C for 30 minutes can also be used. After a rapid wash in 2 × SSC at room temperature and dehydration in graded ethanol series, DAPI in anti-fade solution (0.3 ug DAPI in Vectashield mounting medium, Vector Laboratories) is pipetted onto the specimen and the area is covered with a 20 × 40 mm coverslip. Following this relatively simple protocol, we have found that the FISH assay is successful in a great majority of FFPE specimens (>90% in specimens from large multicenter retrospective studies, ~100% in recently fixed clinical specimens).

We use epifluorescence microscopes equipped with 100 W mercury-arc lamp and fitted with low power (10×) and high power (40× and 100×) high-quality objectives, in combination with single (red, green, blue), dual (red/green) and triple-band pass (red/green/blue) interference filters (Chroma Technology) for simultaneous detection of both EGFR and CEP7 targets in the nuclear context. Digital documentation is secured for two microscope fields (or more in heterogeneous specimens) and a Z-stacking feature is used to account for the multiple focal planes when imaging. Each specimen is evaluated by two independent readers, each of whom analyzes at least 50 cells selected from at least 4 tumor areas. If concordant results are achieved, the result is assigned to the specimen. If there is disagreement, a 3^rd ^reader performs the analysis and the result reached by the majority of the readers is assigned to the specimen.

Lung tumors may be highly heterogeneous with interspaced clusters of tumor cells displaying more or less severe molecular abnormalities, a characteristic that emphasizes the need to comprehensively scan several tumor areas in each section. It is also critical to perform microscope analysis of FISH signals in sections of solid tumors in parallel with the investigation of corresponding H&E stained sections in order to achieve a level of histological interpretation of the section that cannot be provided by the DAPI staining. For practical purposes, the main steps in microscope analysis and interpretation are summarized in Table 1.

**Table 1:** Guidelines for Microscope Analysis of NSCLC tissue sections hybridized with the SpectrumOrange LSI EGFR/SpectrumGreen CEP 7 FISH probe set (Vysis/Abbott Molecular) at UCCC.

• Examine the parallel H&E stained section to locate areas rich in tumor cells. Recognize the tumor pattern, verify the cell density in the tumor areas and the size of the tumor nuclei.
• Identify 4–5 distinct tumor areas and define tissue landmarks for them. Perform this selection with the assistance of a lung pathologist.
• Use low power objective (20× or 40×) and DAPI filter to re-find the selected tumor areas in the FISH section based on the landmarks recognized in the H&E slide. Record the location of these areas.
• Move to a high power objective (100×), change to red, green, double red/green and/or triple blue/red/green band pass filters to inspect quality of the hybridization.
• The normal green signals (CEP 7) signals should be bright, compact (occasionally slightly stringy or diffuse) oval shapes. The red (EGFR) signals should be bright, small round shapes, commonly adjacent to a green CEP 7 signal. The green CEP7 green signal should be larger and brighter than the EGFR red signal.
• Background should appear dark and free of fluorescence particles or haziness.
• At least 75% of cells in the selected tumor areas should display hybridization signals not hampered by background noise for the specimen to qualify for analysis.
• Select approximately 10–20 representative nuclei for analysis in 2–3 microscope fields in each selected tumor area. Record the number of red and green signals for each individual nucleus in the FISH analysis worksheet. Select nuclei should have:
• Not less than median diameter compared with overall tumor nuclei to reduce the effect of the nuclear truncation.
• Unambiguous borders and objectively interpretable signals.
• At least one signal for each target.
• Scan the focus through the entire depth of the section to ensure that all signals are identified within each nucleus.
• Score a minimum of 50 representative nuclei per specimen (or 30 cells when gene amplification is present).
• Document results capturing images of representative fields (two if the specimen is homogeneous or more if the specimen is heterogeneous).

## EGFR FISH patterns and NSCLC patient stratification

The EGFR FISH results obtained in the scored tumor cells are summarized by four indexes representing the (a) percentage of cells carrying at least 4 copies of the EGFR signals, (b) percentage of cells carrying at least 15 copies of the EGFR signals, (c) average ratio of EGFR gene signals/CEP 7 signals per cell, and (d) presence of loose or tight gene clusters or atypically large gene signals. Guidelines for classification of specimens as EGFR FISH positive or negative based on these indexes are listed in Table 2. In short, tumors with at least 40% of cells displaying at least 4 copies of the EGFR signals or with EGFR gene amplification are classified as EGFR FISH positive. Specimens with less than 40% of cells displaying at least 4 copies of the EGFR signals and no gene amplification are classified as EGFR FISH negative.

**Table 2:** UCCC criteria for stratification of NSCLC patients according to the EGFR FISH assay.

**EGFR FISH Positive**
• Specimens with EGFR gene amplification, defined as:
(a) EGFR gene to CEP 7 ratio ≥ 2
(b) Small gene cluster (4–10 copies) or innumerable tight gene cluster in >10% the tumor cells independent of the EGFR to CEP 7 ratio
(c) Larger and brighter EGFR signals than CEP 7 signals in >10% the tumor cells while EGFR signals are smaller than the CEP 7 signals in the adjacent stromal and reactive cells independent of the EGFR to CEP 7 ratio
(d) >15 copies of the EGFR signals in >10% of tumor cells independent of the EGFR to CEP 7 ratio
• Specimens with ≥ 40% of cells displaying ≥ 4 copies of the EGFR signal

**EGFR FISH Negative**
• Specimens without gene amplification as defined above and with <40% of cells displaying ≥ 4 copies of the EGFR signal

The cut-off values for stratification of patients in the EGFR FISH positive or EGFR FISH negative categories were determined in a retrospective study performed in advanced NSCLC patients treated with gefitinib [23]. These patients were initially stratified into six FISH groups according to an increasing number of copies of the EGFR gene in their tumor cells (disomy, low trisomy, high trisomy, low polysomy, high polysomy, and gene amplification) and these groups were evaluated for clinical outcome. The relationship between FISH results and the clinical outcome represented by response to gefitinib, time to disease progression after treatment, and survival after treatment favored the combination of the four FISH groups with no or low genomic gain for EGFR (disomy to low polysomy) as FISH negative and the combination of the two groups with high genomic gain for EGFR (high polysomy and gene amplification) as FISH positive. In that study, among the patients with tumors in the EGFR FISH disomy category there were no responders, 75% of the patients progressed, median time to progression was 2.5 months, and overall survival was 7 months. Similarly poor outcome was noted among the patients with tumors categorized with EGFR FISH trisomy and low polysomy patterns. In contrast, patients with tumors showing EGFR FISH positive pattern included 86% of the responders and only 33% of the patients with progressive disease. Median disease free survival was 6.3 months and overall survival was 9 months in this subset of patients. Although in that retrospective study the response rate was higher and median survival was longer in gefitinib treated patients harboring EGFR gene amplification than high polysomy, differences were not significant and the clinical outcomes in both of these subsets differed significantly from the FISH negative patients.

Similar positive association of EGFR gene copy number with outcome to EGFR-TKIs was found by our UCCC laboratory in three distinct cohorts treated with the EGFR TKI gefitinib [23,32,34] and in an advanced NSCLC cohort treated with erlotinib in which the molecular markers were evaluated at the Princess Margaret Hospital in Toronto, Canada (22). In each of these studies, which included unselected cohorts of patients, the EGFR FISH assay identified a subset of 30–40% of NSCLC patients who were likely to benefit from TKIs. Prospective studies are ongoing for validation of the EGFR FISH assay as a predictive biomarker for EGFR inhibitors. In a preliminary report of the ONCOBELL trial currently being conducted in Italy [41], response rate to gefitinib was 68% in the EGFR-FISH positive and 9% in the EGFR FISH negative NSCLC patients (p < 0.001). Confirmation of the optimal cut-off values in the frequency of tumor cells displaying high gene copy number for identification of EGFR FISH positive patients based on the best fit analysis of clinical outcome to the molecular target therapies is also under way.

Chromogenic in situ hybridization (CISH) is another tissue-based technique with the advantage of using brightfield microscopy [42]. However, CISH has lower level of resolution than FISH and is set for single-color assay therefore preventing the simultaneous use of a control probe for aneusomy. Although we acknowledge the potential applicability of CISH in ascertaining HER2 gene amplification in breast cancer patients who are candidates for trastuzumab therapy, the scoring system proposed for EGFR FISH in NSCLC currently cannot be extrapolated to CISH technology.

## EGFR gene amplification in non-small cell lung carcinoma

**Figure 1:**
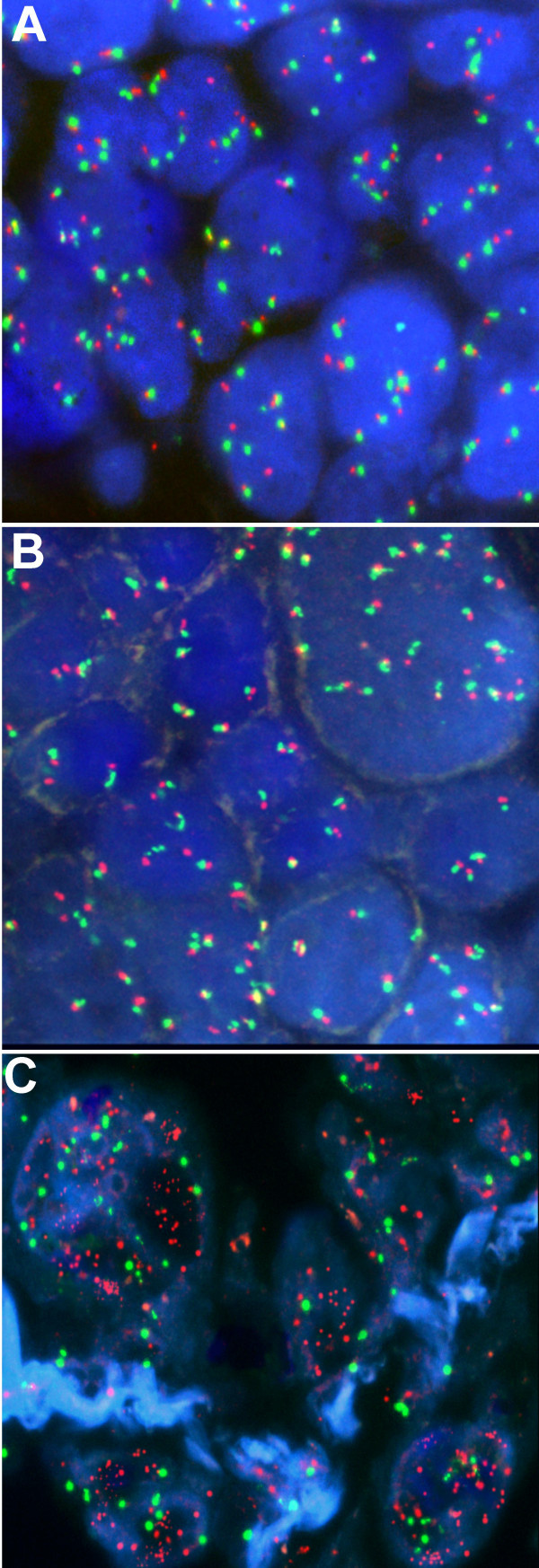
A and B. Examples of advanced non-small cell lung carcinomas showing high level of balanced aneusomy for the EGFR gene and the chromosome 7 centromere probes. C. EGFR gene amplification presented as extrachromosomal double minutes.

**Figure 2:**
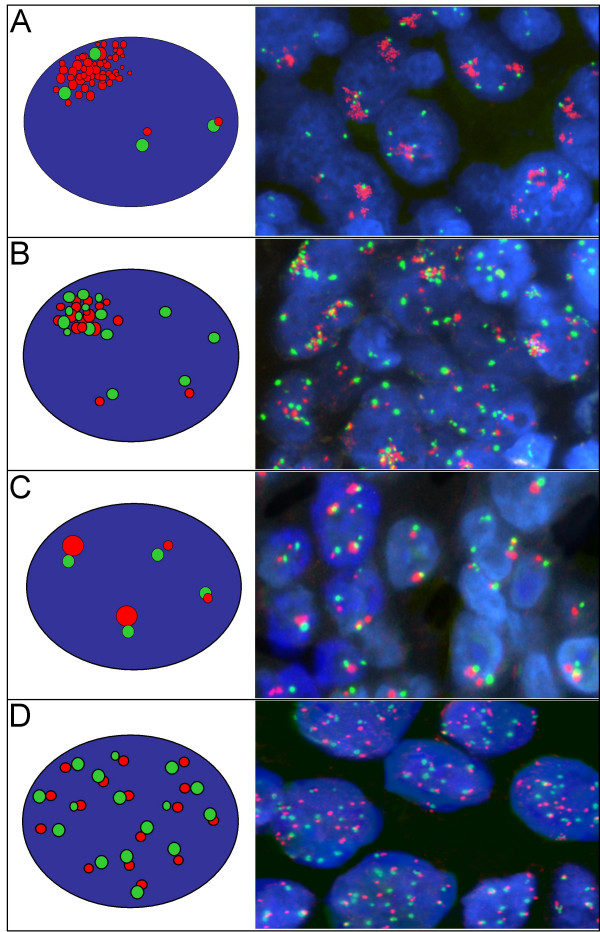
Distinct types of EGFR gene amplification identified in non-small cell lung carcinomas using the EGFR SpectrumOrange/CEP 7 SpectrumGreen FISH probe (Vysis/Abbott Molecular): A. Large EGFR gene clusters; B. Co-localized clusters of EGFR and CEP7 signals; C. Large and bright EGFR signal, larger than the CEP 7 signals in tumor cells; D. High frequency of balanced EGFR and CEP 7 signals.

In breast cancer, amplification of the HER2 gene usually occurs as a large cluster of gene signals surrounding a CEP 17 signal and is classically defined by a ratio of gene to chromosome 17 signals ≥ 2 [3,4]. The classification of EGFR gene amplification in NSCLC is more challenging. One determinant of the complexity is the high frequency of supernumerary copies of CEP 7 (Figure 1A, B) that dilutes the gene gain when only the ratio gene/chromosome is used as an index. Another determinant is the variety of mechanisms of amplification of the EGFR gene. In analyses of large numbers of primary or metastatic NSCLC specimens, we recognized six patterns of EGFR gene amplification, as detailed in Table 3 and illustrated in Figures 1 and 2. The prevalent pattern (~60%) is the classical, large and relatively loose clusters of more than 20 copies of gene signals that is seen when the amplicon is presented as a homogeneously staining region (hsr). In these cases, the ratio EGFR/CEP 7 is likely ≥ 2, despite the usual gain of CEP 7 signals (Figure 2A). A fraction of approximately 10%–15% of amplified tumors displays a second pattern represented by clusters including 4 to 10 copies of EGFR. Combination of these small clusters with extra copies of CEP 7 in each tumor cell usually lowers the ratio EGFR/CEP 7 to less than 2. In the third pattern, found in 15–20% of EGFR amplified cases, the EGFR amplicon includes CEP 7 sequences, which results in co-localized clusters of EGFR and CEP 7 signals (Figure 2B). A fourth pattern is represented by a smaller set of approximately 5% of the amplified cases that display atypically large and bright gene signals most likely representing amplification of a DNA segment encompassed by the EGFR probe (Figure 2C). In the fifth and rarer pattern (~1%) the amplified EGFR sequences are borne on unstable, extrachromosomal double minutes (Figure 1C). The sixth pattern included as gene amplification is a notably high EGFR copy number (≥15 EGFR signals in ≥10% of cells) as consequence of chromosomal aneusomy (Figure 2D), which accounts for approximately 5% of the amplified tumors. Except for the fourth and fifth FISH patterns described above, all others have been similarly observed in metaphase and interphase cells of NSCLC cell lines investigated in our laboratory [43].

**Table 3:** UCCC scoring and interpretation of different types of EGFR gene amplification in NSCLC specimens.

Pattern Description	Scoring Criteria	Number of Cells to Score	Expected Ratio Gene to Chromosome	FISH result*
Large, loose clusters of red (EGFR) signals	Count signals thoroughly and calculate the FISH indexes	30	>2	GA
Small, loose clusters of red (EGFR) signals	Count signals thoroughly and calculate the FISH indexes	30	>2 or ~1 if high level of aneusomy 7 is present	GA if ratio >2 GA if clusters are present in >10% cells, independent of ratio
Large, loose co-localized clusters of red (EGFR) and green (CEP 7) signals	Count signals thoroughly, account for the innumerable signals using the symbol greater than (>) in front of the final counting	30	~1	GA if clusters are present in >10% cells
Tightly packed, innumerable cluster of red (EGFR) signals or atypically large red (EGFR) signal, consistently bigger than the green (CEP 7) signal in the tumor cells but smaller in the adjacent stromal and reactive cells	Count signals in at least 50 cells and identify each cell displaying this specific feature in the analysis sheet	≥ 50	~1	GA if tight clusters or atypical red signal is present in >10% cells
EGFR as double minutes	Count signals thoroughly and calculate the FISH indexes	30	>2	GA
Very high number of balanced red (EGFR) and green (CEP 7) signals	Count signals in at least 50 cells	≥50	~1	GA if >10% cells have ≥ 15 red (EGFR) signals

* GA = EGFR gene amplification

The intratumoral heterogeneity common in NSCLC adds to the described complexity. EGFR gene amplification may be homogenously distributed over the tumor areas in a tissue section or may be confined to certain cells. In this case, it may be present in specific foci of tumor cells or diffusely interspaced among non-amplified tumor nuclei. These findings have supported the definition of EGFR amplification in lung tumors in our studies based on a minimum percentage of cells (≥10%) instead of using the gene to chromosome ratio >2 classically accepted and approved by the US Food and Drug Administration (FDA) for the HER2 gene in breast cancer. In the heterogeneous specimens, the ratio EGFR to CEP 7 can be significantly impacted by the selection of cells to be scored, thus sustaining the proposed scoring in multiple tumor areas as an attempt to obtain a more representative result.

## Conclusion

The EGFR FISH assay according to the described scoring criteria developed in our laboratory at the University of Colorado Cancer Center allows for the assessment of EGFR gene patterns that can assist in the stratification of advanced NSCLC patients for treatment with the EGFR-TKIs gefitinib and erlotinib. The assay is readily applicable to thin sections of standard FFPE tumor blocks and results can be determined in nuclei selected within the correct histological context. However, we acknowledge that additional information from prospective clinical trials is still required before recommendations relating to patient management can be made. Studies combining EGFR FISH with other molecular markers such as EGFR IHC and mutation analyses are also ongoing, and it is possible that a panel of tests ultimately prove to be better than any single test for NSCLC patient selection for TKI.

The utility of the FISH assay in FFPE sections is expected to broaden in the future as additional chromosomal and genomic abnormalities are identified as prognostic and predictive molecular markers in human cancers. Numerous solid tumors have been considered for molecular targeted therapies and selection of patients for those treatments will very likely be dependent on the tumor molecular profile. It is critical to define guidelines for laboratorial assays and interpretation of the results for each specific case and to put in place successful quality control and quality assurance programs to qualify clinical laboratories to offer these tests.

## Competing interests

In the last 5 years, Marileila Varella-Garcia received honorarium from Abbott Molecular of <$2,000. Marileila Varella-Garcia is one of the Inventors/Applicants for the Patent Application PCT/US2005/018879; Title: Methods for Prediction of Clinical Outcome to Epidermal Growth Factor Receptor Inhibitor by Cancer Patients (University of Colorado).
